# Multiple Streams Analysis of the Long-Term Care Insurance in South Korea: Understanding Policy Changes (2008 - 2014)

**DOI:** 10.1093/geroni/igab046.3102

**Published:** 2021-12-17

**Authors:** Mijin Jeong

**Affiliations:** University of Kansas, LAWRENCE, Kansas, United States

## Abstract

The Long-Term Care Insurance (LTCI) Act in South Korea was enacted in 2008 to improve the quality of life of older adults by promoting better health and to mitigate the burden of care on family members. In 2014, the Enforcement Decree for the LTCI Act was revised to broaden criteria for eligible recipients of LTCI-related services and care. This policy analysis seeks to explore the political circumstances under which the Act was formed and how social environmental factors had evolved to revise the LTCI Act using a multiple streams policy analysis framework. A combination of factors influenced the status of LTCI policy agenda, including shifts in aged demographic structure and increasing medical expenditures. From the Korean National Dementia Plan, a pilot project of dementia care was conducting to prove the efficiency of dementia care service. While the Korean Senior Citizens Association (KSCA) was less successful gaining press attention around dementia care, the presidential election and candidates’ election pledges were key factors to suddenly open the opportunity to extend the recipients for dementia care. The process through which the LTCI Act was revised and expanded showed the importance of the political environment associated with the election. Based on the recognition of LTIC policy agenda and already testing the efficiency of dementia care services, the election leaded to revision of LTCI Act and it quickly diffused by the new administration. From the revision of LTCI, international policymakers and scholars should recognize how the political events might use the policy for older adutls.

